# Effects of *Moringa oleifera* Oil on Adipokine Responses, Inflammation, Oxidative Stress, Bacterial Burden, and Early Survival in CLP-Induced Septic Rats

**DOI:** 10.3390/antiox15091128

**Published:** 2026-09-07

**Authors:** Ufuk Ülker, Bülent Bayraktar, Mehmet Eray Alçığır, Sibel Kızıl, Tünay Karan, Gökşad Cemil Kotan, Mustafa Yeni

**Affiliations:** 1 Department of Veterinary Microbiology, Faculty of Veterinary Medicine, Yozgat Bozok University, Yozgat 66100, Türkiye; ufuk.ulker@bozok.edu.tr; 2 Department of Physiotherapy and Rehabilitation, Faculty of Health Sciences, Bayburt University, Bayburt 69000, Türkiye; bulentbayraktar@bayburt.edu.tr; 3 Department of Veterinary Pathology, Faculty of Veterinary Medicine, Kırıkkale University, Yahşihan 71450, Türkiye; erayalcigir@kku.edu.tr; 4 Department of Veterinary Microbiology, Faculty of Veterinary Medicine, Kırıkkale University, Yahşihan 71450, Türkiye; sibelkizil@kku.edu.tr; 5 Department of Veterinary Genetics, Faculty of Veterinary Medicine, Yozgat Bozok University, Yozgat 66100, Türkiye; tunay.karan@bozok.edu.tr; 6 Laboratory and Veterinary Health Program, Department of Veterinary Medicine, Alaca Avni Çelik Vocational School, Hitit University, Alaca 19600, Türkiye; goksadcemilkotan@hitit.edu.tr; 7 Erzurum Regional Training and Research Hospital, Yakutiye 25240, Türkiye

**Keywords:** sepsis, cecal ligation and puncture, *Moringa oleifera*, adipokines, oxidative stress, inflammation, bacterial burden, good health and well-being (SDG 3)

## Abstract

*Moringa oleifera* has anti-inflammatory and antioxidant properties, but its effects in polymicrobial sepsis are incompletely defined. We evaluated M. oleifera oil (MOO) in female Wistar rats subjected to cecal ligation and puncture (CLP). Forty rats were randomized to Control, Sham, CLP, CLP + MOO 100 mg/kg, or CLP + MOO 200 mg/kg (*n* = 8/group). At 24 h, survival, clinical scores, serum apelin, omentin-1, interleukin-6 (IL-6), tumor necrosis factor-α (TNF-α), malondialdehyde (MDA), recoverable bacterial burden, and histopathology were assessed. CLP decreased omentin-1 and increased IL-6, TNF-α, MDA, recoverable *Staphylococcus aureus* and *Escherichia coli*, clinical severity, and multi-organ injury. At 200 mg/kg, apelin and omentin-1 were 1.15 ± 0.12 and 28.4 ± 3.1 ng/mL, while IL-6, TNF-α, and MDA decreased to 18.4 ± 1.8 pg/mL, 24.1 ± 2.8 pg/mL, and 2.5 ± 0.3 nmol/mL, respectively. Neither organism was recovered above the detection limit. Survival was 62.5% in CLP, 87.5% with 100 mg/kg, and 100% with 200 mg/kg MOO. Within this 24 h model, a single post-CLP oral dose of MOO was associated with lower inflammatory cytokine concentrations and MDA, reduced recoverable bacterial burden, improved clinical and histopathological findings, and higher descriptive survival. Because MDA was the sole oxidative endpoint and no non-septic MOO-only group or molecular redox assays were included, these findings support the attenuation of sepsis-associated lipid peroxidation but do not establish a direct antioxidant mechanism.

## 1. Introduction

Sepsis is a life-threatening systemic organ dysfunction caused by a dysregulated host response to infection [1]. It represents a complex pathological process that develops following pathogen invasion and may involve cytokine dysregulation, capillary leakage, microcirculatory disturbances, and multiple organ failure [2]. Tissue injury in sepsis is largely associated with impaired perfusion and disrupted cellular oxygen utilization [3]. Disruption of the alveolar–endothelial barrier may lead to acute respiratory distress syndrome, reduced renal perfusion may result in acute kidney injury, and impairment of the blood–brain barrier may contribute to severe complications such as septic encephalopathy [4]. The role of adipokines in regulating inflammatory and metabolic responses during sepsis has attracted increasing attention [5]. Apelin is an endogenous peptide that acts through the APJ receptor and contributes to the regulation of cardiovascular homeostasis, vascular tone, fluid balance, and energy metabolism [6,7]. Experimental studies suggest that the Apelin system may attenuate sepsis-associated myocardial dysfunction and inflammatory injury [8,9]. However, circulating Apelin levels during sepsis may vary depending on the experimental model, disease stage, age, and sampling time [8,10]. Omentin-1 is an adipokine primarily secreted by visceral adipose tissue and is associated with anti-inflammatory, metabolic regulatory, and endothelial-protective effects [11]. Clinical findings regarding Omentin-1 levels in sepsis remain inconsistent. Gao et al. [12] reported that serum Omentin-1 levels decreased with increasing sepsis severity, whereas Karampela et al. [13] showed that elevated Omentin-1 levels may be associated with septic shock and mortality. The omentin-1 response varies depending on disease stage, metabolic status, and compensatory host responses [5]. In sepsis, increased levels of pro-inflammatory cytokines such as IL-6 and TNF-α are associated with endothelial injury, increased vascular permeability, and organ dysfunction [14,15]. In parallel, excessive production of reactive oxygen species and insufficient antioxidant defense promote lipid peroxidation, while malondialdehyde is widely used as a marker of oxidative damage [16,17]. Therefore, reducing bacterial burden while simultaneously limiting inflammatory and oxidative injury represents an important objective in experimental sepsis interventions [18].

*Moringa oleifera* contains various bioactive constituents, including isothiocyanates, flavonoids, and phenolic compounds [19]. Isothiocyanate-rich Moringa extracts have been reported to suppress NF-κB-related pro-inflammatory responses and support Nrf2-mediated antioxidant defense [20,21]. Sailaja et al. [21] demonstrated that Moringa isothiocyanate-1 reduced TNF-α, IL-1β, and IL-6 expression and enhanced Nrf2 activation in an LPS-induced inflammatory model. Nevertheless, the combined effects of *Moringa oleifera* oil on Apelin and Omentin-1 responses, bacterial burden, early survival, and multiple-organ injury in CLP-induced sepsis remain insufficiently characterized. Therefore, the present study evaluated the effects of *Moringa oleifera* oil administered at doses of 100 and 200 mg/kg in a rat model of CLP-induced sepsis. Serum Apelin, Omentin-1, IL-6, TNF-α, and MDA levels; recoverable *Staphylococcus aureus* and *Escherichia coli* burdens; clinical signs of sepsis; 24 h survival; and histopathological changes in the heart, lungs, liver, and kidneys were assessed.

## 2. Materials and Methods

### 2.1. Animals and Experimental Groups

A total of 40 adult female Wistar albino rats with initial body weights ranging from 180 to 200 g were used in the study. The animals were housed under standard laboratory conditions at 21–24 °C and 40–45% relative humidity, with a 12 h light/12 h dark cycle, and were provided with fresh water and standard pellet feed ad libitum. The rats were randomized to ensure comparable mean baseline body weights and were allocated into five experimental groups, each containing eight animals (*n* = 8), as follows:

Only female rats were included. The experiment was not designed to evaluate sex effects, and no a priori sex-specific biological rationale was documented. Accordingly, the results should not be generalized to male animals; future studies should include both sexes and prespecified sex-stratified analyses.

Control group (C): Animals that received no surgical or pharmacological intervention and were used to determine healthy baseline values.

Sham group (S): Animals that underwent laparotomy only, without sepsis induction, to evaluate the effects of surgical stress on the measured parameters.

Sepsis group (CLP): Animals in which sepsis was induced by the cecal ligation and puncture method, without any therapeutic intervention; this group served as the negative control.

CLP + MOO 100 group: Animals received a single 100 mg/kg oral dose of *Moringa oleifera* oil following sepsis induction; no repeat dose was administered during the 24 h follow-up.

CLP + MOO 200 group: Animals received a single 200 mg/kg oral dose of *Moringa oleifera* oil following sepsis induction; no repeat dose was administered during the 24 h follow-up.

**Figure 1:**
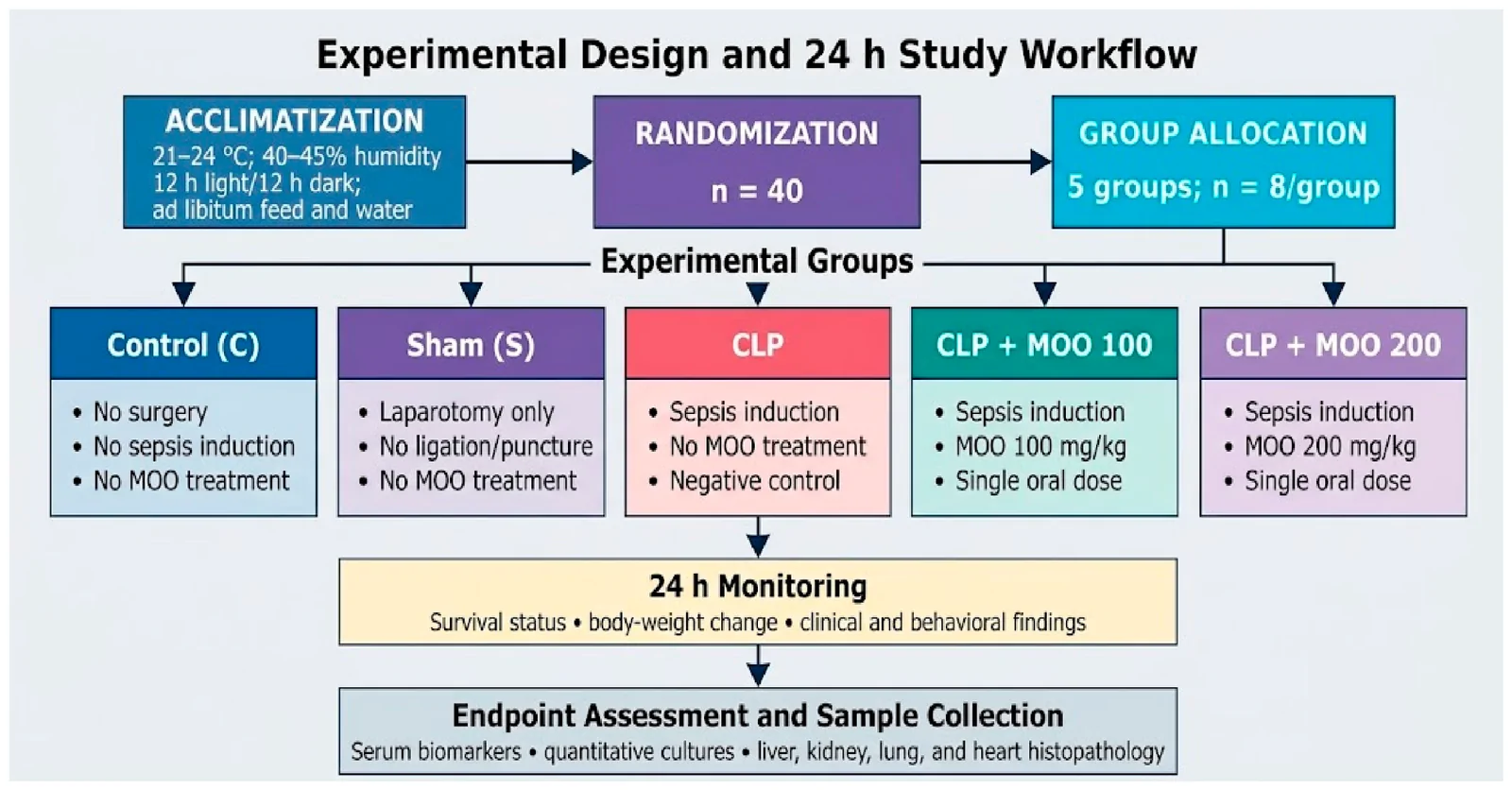
A schematic representation of the experimental design, animal groups, and 24 h workflow used to evaluate *Moringa oleifera* oil in CLP-induced sepsis. CLP, cecal ligation and puncture; MOO, *Moringa oleifera* oil. MOO was given once after CLP induction; no repeat dose was administered during follow-up.

The experimental protocol was approved by the Local Ethics Committee for Animal Experiments of Ankara Training and Research Hospital, University of Health Sciences, Republic of Türkiye Ministry of Health (Meeting No: 0087; Decision No: 818; approval date: 16 January 2025). During the 24 h follow-up period, sepsis-related changes in body weight and survival status were monitored and recorded simultaneously in all groups. The experimental workflow is summarized in Figure 1.

### 2.2. Induction of Sepsis (CLP Model)

Sepsis was induced using the Cecal Ligation and Puncture (CLP) model, which is widely recognized as the gold standard for experimental polymicrobial sepsis. Following the administration of systemic anesthesia consisting of 10 mg/kg xylazine and 50 mg/kg ketamine, a 3 cm midline laparotomy was performed under aseptic conditions. The cecum was carefully exteriorized and ligated with 4/0 silk at a position distal to the ileocecal valve to maintain intestinal continuity [22,23]. To initiate the septic insult, the ligated cecal stump was perforated twice using an 18-gauge needle, and a small amount of fecal material was gently extruded to ensure patency and a consistent polymicrobial release into the peritoneal cavity. The cecum was then repositioned into its anatomical location within the abdominal cavity, and the incision was closed in two distinct layers using standard surgical sutures. Sham-operated rats underwent the identical surgical laparotomy and cecal manipulation but were not subjected to ligation or perforation, thereby serving as a control for surgical stress.

### 2.3. Moringa oleifera Oil: Material Documentation and Chemical Characterization

The intervention is designated *Moringa oleifera* oil (MOO), rather than the previous imprecise label, because it was administered as a lipid-rich oil. The retained experimental records did not document the plant part used to produce the oil, its geographical or commercial origin, botanical authenticator or voucher specimen, batch/lot number, or extraction/manufacturing procedure. Extraction was not performed as part of the present animal experiment. To avoid introducing unverifiable information, no seed- or leaf-origin or extraction method claim is made. The submitted oil sample was chemically characterized according to ASU §64 LFGB L 07.00-40 [24] using HPLC-UV/FLD. Total fat was 98.7 g/100 g; the fatty acid classes comprised 74.2 g/100 g monounsaturated, 22.2 g/100 g saturated, and 3.2 g/100 g polyunsaturated fatty acids. Vitamin E and lutein were measured at 21 mg/100 g and 0.25 mg/100 g, respectively, whereas vitamins A and D3 and the carotenoids zeaxanthin, β-carotene, and lycopene were present at trace levels. The analysis also detected naphthalene (23 µg/kg), phenanthrene (8.5 µg/kg), and fluorene (4.4 µg/kg), with pyrene, fluoranthene, acenaphthene, and anthracene also reported. These polycyclic aromatic hydrocarbons are treated as potential environmental or processing-related contaminants, not as putative bioactive constituents.

### 2.4. Treatment Procedure

*Moringa oleifera* oil (MOO) was administered once to the treatment groups at 100 or 200 mg/kg by oral gavage following completion of the CLP procedure. No repeat dose was administered during the subsequent 24 h follow-up. Survival status, body weight change, and clinical sepsis indicators (lethargy, piloerection, and rectal temperature) were monitored throughout the follow-up.

### 2.5. Measurement of Serum Biomarkers

Quantification of the target parameters was performed using rat-specific commercial ELISA kits. Serum Apelin (C12ORF39) concentrations were measured using a kit with a detection range of 2–600 pg/mL (Shanghai YL Biotech Co., Ltd. [YL Biont], Shanghai, China; Cat. No. YLA1681RA), whereas Omentin-1 (ITLN1) concentrations were determined using a kit with a measurement range of 2–600 ng/L (Shanghai YL Biotech Co., Ltd., Shanghai, China; Cat. No. YLA0197RA). Interleukin-6 (IL-6) levels were measured within a range of 8–150 ng/L using a rat-specific ELISA kit (SinoGeneClon Biotech Co., Ltd., Hangzhou, China; Cat. No. SG-20267). TNF-α levels were quantified using the corresponding rat-specific ELISA kit (SinoGeneClon Biotech Co., Ltd., Hangzhou, China; Cat. No. SG-20127) in accordance with the manufacturer’s instructions. Serum malondialdehyde (MDA) levels were measured using a rat-specific commercial ELISA kit (SinoGeneClon Biotech Co., Ltd., Hangzhou, China; Cat. No. SG-20889) according to the manufacturer’s protocol. Optical density was measured at 450 nm using an Epoch microplate spectrophotometer (BioTek Instruments, Inc., Winooski, VT, USA), and analyte concentrations were calculated from the corresponding standard curves. MDA results were expressed as nmol/mL.

### 2.6. Quantitative Microbiological Analysis

To assess the extent of systemic infection and bacterial clearance, blood and peritoneal fluid samples were collected under aseptic conditions and inoculated onto nutrient agar, blood agar, and MacConkey agar. Following incubation at 37 °C for 24 h, quantitative colony counts were performed and expressed as log10 CFU/mL. Presumptive identification of *Staphylococcus aureus* and *Escherichia coli* was based on colony morphology, Gram staining, and appropriate biochemical confirmation tests conducted according to standard microbiological procedures.

### 2.7. Histopathological Examination

To evaluate systemic organ injury and tissue integrity at the microscopic level, liver, lung, kidney, and heart tissues were fixed in 10% neutral buffered formalin for 48 h. Following fixation, the samples were processed using routine histological procedures, embedded in paraffin, sectioned at a thickness of 5 µm, and stained with hematoxylin and eosin (H&E). The prepared sections were examined under a light microscope for parenchymal degeneration, inflammatory cell infiltration, necrotic foci, and vascular alterations. Histopathological damage was graded using the following semiquantitative scoring system: (−), 0–10%, absent or minimal; (+), 10–30%, mild; (++), 30–50%, moderate; and (+++), >50%, severe. Representative spleen sections were also included in the photomicrographic panel; however, the predefined semiquantitative injury scoring was restricted to liver, kidney, lung, and heart tissues.

### 2.8. Statistical Analysis

Statistical analyses were performed using IBM SPSS Statistics, version 26.0 (IBM Corp., Armonk, NY, USA). Continuous variables are presented as the mean ± standard deviation. Differences among groups were analyzed using one-way analysis of variance, followed by Duncan’s multiple range test for post hoc comparisons. Survival status at 24 h was presented descriptively as the number and percentage of surviving animals in each group. A *p*-value of less than 0.05 was considered statistically significant.

The retained revision records contained group-level summary values (mean ± SD) rather than verifiable individual-animal observations. Individual-animal data scatter plots were therefore not generated, because reconstructing apparent observations from summary statistics would be scientifically misleading.

## 3. Results

### 3.1. Biochemical Findings

In this study, the effects of *Moringa oleifera* oil (MOO) were evaluated in a rat model of CLP-induced sepsis by assessing adipokine-related biomarkers, inflammatory cytokines, oxidative stress status, recoverable bacterial burden, clinical findings, 24 h survival, and histopathological changes in multiple organs.

CLP-induced sepsis caused marked alterations in the measured biochemical parameters. Serum Apelin levels were comparable among the Control, Sham, and CLP groups, with values of 0.45 ± 0.04 ng/mL, 0.43 ± 0.05 ng/mL, and 0.42 ± 0.05 ng/mL, respectively. In contrast, Omentin-1 levels were substantially reduced in the CLP group compared with the Control and Sham groups. The Control and Sham groups showed Omentin-1 levels of 32.1 ± 2.5 ng/mL and 31.8 ± 2.2 ng/mL, respectively, whereas the CLP group showed a lower value of 12.4 ± 1.5 ng/mL. The induction of sepsis also resulted in a pronounced increase in pro-inflammatory and oxidative stress markers. IL-6 levels increased from 12.2 ± 1.1 pg/mL in the Control group and 12.8 ± 1.3 pg/mL in the Sham group to 45.2 ± 3.1 pg/mL in the CLP group. Similarly, TNF-α levels increased to 58.6 ± 4.2 pg/mL in the CLP group, compared with 14.8 ± 1.5 pg/mL and 15.5 ± 1.7 pg/mL in the Control and Sham groups, respectively. MDA levels, used as an indicator of lipid peroxidation, were also highest in the CLP group, reaching 6.8 ± 0.5 nmol/mL, whereas the Control and Sham groups showed lower values of 1.7 ± 0.2 nmol/mL and 1.9 ± 0.3 nmol/mL, respectively. Both MOO doses were associated with improvement, and the 200 mg/kg dose produced greater changes than the 100 mg/kg dose under the tested conditions. In the CLP + MOO 100 mg/kg group, Apelin increased to 0.68 ± 0.08 ng/mL and Omentin-1 increased to 18.6 ± 2.2 ng/mL. In the same group, IL-6, TNF-α, and MDA levels decreased to 32.1 ± 2.4 pg/mL, 42.3 ± 3.5 pg/mL, and 4.2 ± 0.4 nmol/mL, respectively. These findings indicate a partial improvement compared with the untreated CLP group. The most pronounced biochemical changes were observed in the CLP + MOO 200 mg/kg group. In this group, Apelin levels increased to 1.15 ± 0.12 ng/mL, exceeding the values observed in the Control and Sham groups. Omentin-1 levels also increased to 28.4 ± 3.1 ng/mL, approaching the values of the non-septic groups. In parallel, IL-6 decreased to 18.4 ± 1.8 pg/mL, TNF-α decreased to 24.1 ± 2.8 pg/mL, and MDA decreased to 2.5 ± 0.3 nmol/mL. Overall group differences were statistically significant for all measured biochemical parameters (*p* < 0.001; Table 1).

### 3.2. Recoverable Bacterial Burden

Quantitative microbiological analysis showed that recoverable bacterial counts were highest in the untreated CLP group. No bacterial growth was detected in the Control group. In the Sham group, *S. aureus* positivity was observed in 12.5% of samples, with a recoverable bacterial count of 1.15 ± 0.30 log10 CFU/mL, whereas *E. coli* was not recovered above the detection limit. In the untreated CLP group, *S. aureus* positivity reached 87.5%, with a recoverable count of 1.69 ± 0.85 log10 CFU/mL. *E. coli* positivity was 75%, with a recoverable count of 2.10 ± 0.45 log10 CFU/mL. These findings indicate that the CLP procedure produced a marked polymicrobial infectious burden. MOO administration was associated with a reduction in recoverable bacterial counts. In the CLP + MOO 100 mg/kg group, *S. aureus* positivity decreased to 12.5%, and recoverable *S. aureus* counts were below 1.00 log10 CFU/mL. In the same group, *E. coli* was not recovered above the detection limit. In the CLP + MOO 200 mg/kg group, neither *S. aureus* nor *E. coli* was recovered above the detection limit. These findings suggest that MOO treatment was associated with a marked reduction in recoverable bacterial burden in septic rats. However, because direct antibacterial assays such as minimum inhibitory concentration, minimum bactericidal concentration, time-kill analysis, or phagocytic activity assays were not performed, these findings should not be interpreted as direct evidence of bactericidal activity. Rather, they indicate reduced bacterial recovery under the conditions of the present experimental model (Table 2).

### 3.3. Clinical Findings and 24 h Survival

Clinical observations supported the biochemical and microbiological findings. The Control group displayed normal behavior, normal stool consistency, no pain response, and a clinical score of 0.0 ± 0.0. The Sham group also showed near-normal clinical findings, with a clinical score of 0.2 ± 0.1 and 100% survival. In contrast, the untreated CLP group developed severe clinical signs compatible with systemic sepsis. These animals showed marked lethargy, reduced activity, piloerection, diarrhea, and increased pain response. The behavioral score in the CLP group was recorded as 4, corresponding to severe sepsis, and the clinical score increased to 3.8 ± 0.5. The Grimace pain score was 3, and stool consistency was diarrheic. At the end of the 24 h observation period, survival in the CLP group was 5/8 animals, corresponding to a survival rate of 62.5%. MOO treatment was associated with improvement in clinical and behavioral parameters. In the CLP + MOO 100 mg/kg group, the behavioral score decreased to 2, indicating mild lethargy, and the clinical score decreased to 1.8 ± 0.3. The Grimace pain score was 2, stool consistency was soft, and 7/8 animals survived during the 24 h observation period, corresponding to a survival rate of 87.5%. In the CLP + MOO 200 mg/kg group, clinical improvement was more pronounced. The behavioral score decreased to 1, indicating an active state, and the clinical score decreased to 1.0 ± 0.2. The Grimace pain score was reduced to 1, and stool consistency was recorded as normal. All animals in this group survived during the 24 h observation period, with a survival rate of 8/8 animals, corresponding to 100%. Survival data were presented descriptively as the number and percentage of surviving animals in each group, without Kaplan–Meier analysis, because individual death times were not evaluated (Table 3).

### 3.4. Histopathological Findings

**Figure 2:**
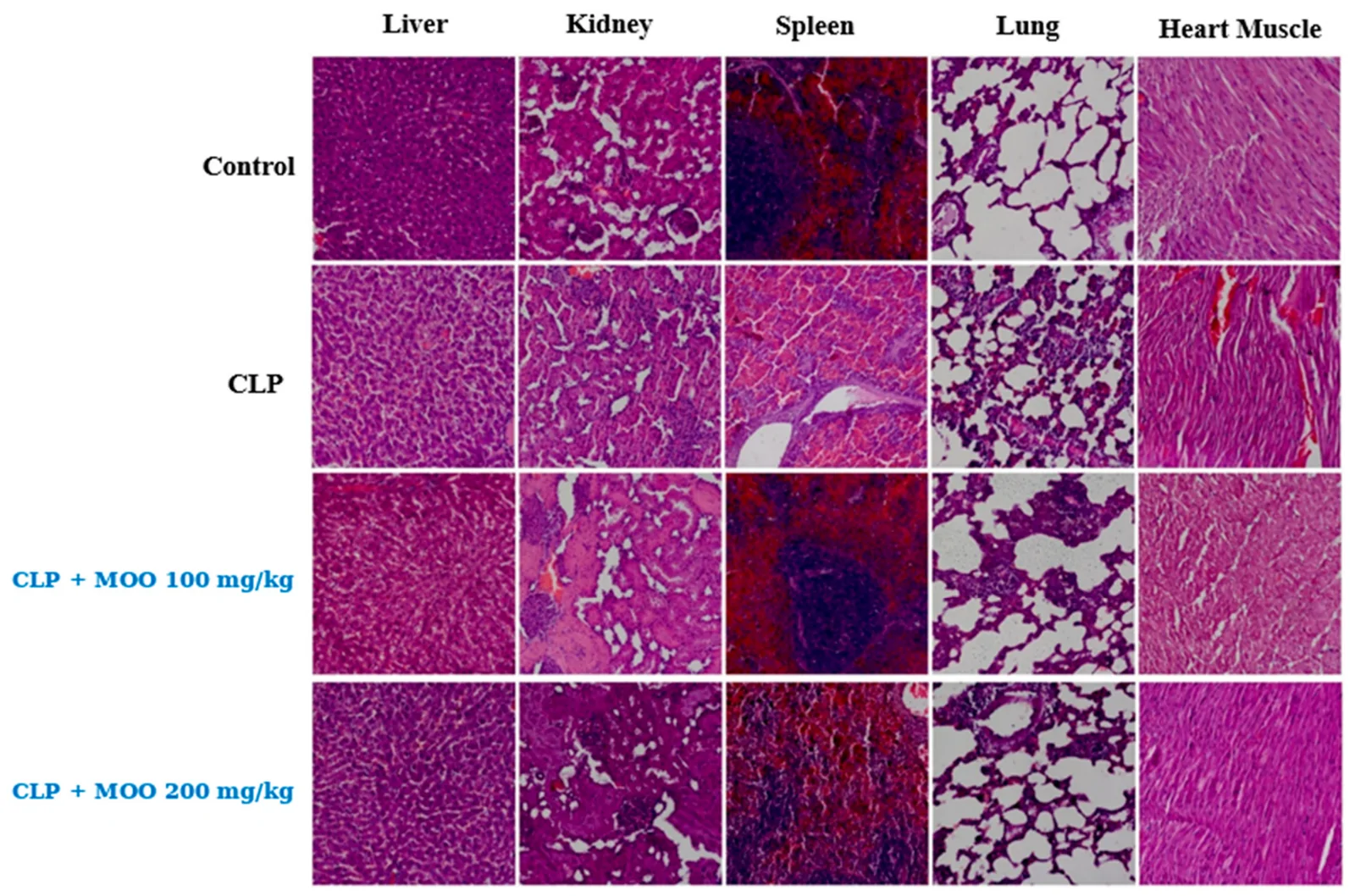
Representative hematoxylin and eosin-stained sections of liver, kidney, spleen, lung, and heart tissues from the Control, CLP, CLP + MOO 100 mg/kg, and CLP + MOO 200 mg/kg groups. Columns show the indicated tissues, and rows show the indicated experimental groups. The spleen was included in the representative photomicrographic panel but was not part of the predefined semiquantitative injury scoring reported in Table 4. CLP, cecal ligation and puncture; MOO, *Moringa oleifera* oil. The archived photomicrographic montage did not retain pixel-to-micrometer calibration metadata; consequently, a valid scale bar could not be reconstructed retrospectively, and the panel should be interpreted as a qualitative representative comparison.

Histopathological examination revealed preserved tissue architecture in the Control and Sham groups. In these groups, liver, kidney, lung, and heart tissues generally showed normal histological appearance, with no prominent parenchymal degeneration, necrotic foci, or severe inflammatory cell infiltration. In the untreated CLP group, sepsis caused marked histopathological injury in multiple organs. Liver tissue showed prominent parenchymal degeneration, vacuolar changes, and inflammatory cell infiltration. Kidney sections demonstrated tubular epithelial injury, vacuolar degeneration, and vascular alterations compatible with sepsis-related renal damage. Lung tissue showed inflammatory cell infiltration and vascular changes, suggesting acute inflammatory involvement. In heart tissue, parenchymal degeneration and inflammatory alterations were observed, indicating sepsis-associated myocardial injury. MOO administration was associated with attenuation of these histopathological lesions, particularly at the higher dose. In the CLP + MOO 100 mg/kg group, partial improvement was observed in several tissues compared with the untreated CLP group; however, inflammatory and degenerative findings were still evident in some organs. In the CLP + MOO 200 mg/kg group, tissue architecture was better preserved, and inflammatory infiltration, vascular alterations, and degenerative changes appeared less prominent than in the untreated CLP group. These findings indicate that high-dose MOO treatment was associated with reduced sepsis-related histopathological injury in the liver, kidney, lung, and heart. Overall, the histopathological findings were consistent with the biochemical, microbiological, and clinical results. The reduction in IL-6, TNF-α, and MDA levels, together with decreased recoverable bacterial burden and improved clinical scores, was accompanied by less severe tissue injury in the MOO-treated groups, especially in the CLP + MOO 200 mg/kg group. Taken together, CLP-induced sepsis resulted in increased inflammatory and oxidative stress markers, reduced Omentin-1 levels, high recoverable bacterial burden, severe clinical findings, reduced 24 h survival, and marked multi-organ histopathological injury. MOO administration, particularly at 200 mg/kg, was associated with increased Apelin and Omentin-1 levels, decreased IL-6, TNF-α, and MDA levels, reduced recoverable *S. aureus* and *E. coli* counts, improved clinical and behavioral scores, higher 24 h survival, and attenuation of histopathological damage. These findings suggest that MOO may exert multi-target protective effects in CLP-induced experimental sepsis, although direct bactericidal activity and specific molecular mechanisms require further investigation. Representative sections are shown in Figure 2.

**Table 4:** Semiquantitative histopathological injury scores in liver, kidney, lung, and heart tissues.

Treatment Group	Liver	Kidney	Lung	Heart
Control	−	−	−	−
Sham	−	−	−	−
CLP	+++	+++	++	++
CLP + MOO 100 mg/kg	++	++	+	+
CLP + MOO 200 mg/kg	+	+	−/+	−/+

Histopathological injury was scored semiquantitatively as follows: −, absent or minimal injury; +, mild injury; ++, moderate injury; +++, severe injury. CLP, cecal ligation and puncture; MOO, *Moringa oleifera* oil.

## 4. Discussion

### 4.1. Adipokine Responses

Sepsis is a complex clinical condition in which the systemic inflammatory response becomes dysregulated and leads to life-threatening organ dysfunction [25,26]. Apelin is an endogenous peptide with anti-inflammatory properties that may suppress reactive oxygen species through the AMPK/NOX4 axis and preserve VE cadherin integrity, thereby limiting microvascular leakage in sepsis [27]. It also contributes to cardioprotection by increasing myocardial contractility through calcium sensitization independently of the adrenergic system [28,29]. In addition, Apelin may function as a multisystem cytoprotective mediator against excessive inflammatory activation by suppressing NF-κB and NLRP3 inflammasome pathways [9,29]. Cao et al. [9] reported that Apelin activates the AMPK signaling pathway in sepsis, inhibits NLRP3-mediated pyroptosis, and thereby attenuates myocardial dysfunction.

In the present study, serum Apelin levels in the CLP group were lower than those in the Control group, with a value of 0.42 ± 0.05 ng/mL, and was higher in the 200 mg/kg group than in the 100 mg/kg group under the tested conditions. Administration of 200 mg/kg MOO increased serum Apelin levels to 1.15 ± 0.12 ng/mL, exceeding basal values and indicating a marked neurometabolic regulatory response. Our results are consistent with previous studies reporting decreased serum Apelin levels in CLP-induced sepsis models [8], whereas they differ from studies reporting increased Apelin levels in septic cases [30,31]. This discrepancy may be related to differences in experimental model, sampling time, disease stage, age, and compensatory host responses.

The greater increase in Apelin at 200 mg/kg than at 100 mg/kg under the tested conditions is a novel treatment-associated finding, but it does not by itself establish restoration of a defined Apelin-dependent pathway. The study did not measure tissue Apelin/APJ expression, AMPK/NOX4 signaling, NF-κB or Nrf2 activation, or downstream endothelial barrier targets. Accordingly, the Apelin result is interpreted as an association that accompanies the broader biochemical and histological improvement, rather than proof that Apelin mediates the effect of MOO.

Omentin-1 is an anti-inflammatory adipokine involved in the suppression of pro-inflammatory cytokine release and endothelial injury in inflammatory conditions, including sepsis [32]. In the present study, serum Omentin-1 levels were significantly reduced in the CLP group (12.4 ± 1.5 ng/mL). This finding is consistent with the results of Gao et al. [12], who reported that Omentin-1 levels decreased progressively with increasing sepsis severity and showed a negative association with APACHE II scores. In contrast, Karampela et al. [13] reported increased Omentin-1 levels at the onset of sepsis. The difference between their findings and the present results may be related to differences in sampling time, disease stage, clinical severity, and the compensatory dynamics of adipokine responses during sepsis. In the present study, Omentin-1 was higher after MOO treatment, with a greater increase at 200 mg/kg than at 100 mg/kg under the tested conditions, with the 200 mg/kg dose increasing serum Omentin-1 to 28.4 ± 3.1 ng/mL. This value approached the levels observed in the non-septic groups and suggests that MOO may contribute to the restoration of suppressed adipokine responses during sepsis. At the molecular level, the reported ability of Omentin-1 to activate the Akt/eNOS pathway and suppress NF-κB activation and pro-inflammatory cytokine synthesis may help explain the reduction in IL-6 and TNF-α levels observed in the MOO-treated groups. Similarly, studies by Qi et al. [33] and Zhou et al. [34] have shown that Omentin-1 can reduce oxidative stress by suppressing ROS production. In line with these findings, the elevated MDA level observed in the CLP group (6.8 ± 0.5 nmol/mL) decreased to 2.5 ± 0.3 nmol/mL in the CLP + MOO 200 mg/kg group. Therefore, the MOO-associated increase in Omentin-1 was accompanied by reduced inflammatory and oxidative stress markers, improved clinical findings, and reduced recoverable bacterial burden, supporting the possible therapeutic relevance of MOO in experimental sepsis.

### 4.2. Inflammatory and Redox Findings in Relation to Oil Composition

In sepsis, excessive cytokine signaling and oxidative injury contribute to multiple-organ dysfunction [35]. In the present study, CLP increased IL-6, TNF-α, and MDA, whereas both MOO doses were associated with lower values and the 200 mg/kg dose produced the largest reductions. The MDA result is compatible with attenuation of lipid peroxidation during the 24 h experiment; however, MDA was the only redox endpoint measured. Because endogenous antioxidant enzymes, glutathione status, reactive oxygen species, and redox-sensitive signaling pathways were not assessed, the data do not establish a complete antioxidant mechanism or the intrinsic antioxidant effect of MOO outside sepsis.

The analytical profile of the administered MOO provides a biologically plausible, but not constituent-specific, context for these findings. The sample contained 98.7 g/100 g total fat and was dominated by monounsaturated fatty acids (74.2 g/100 g), with lower saturated and polyunsaturated fractions, together with vitamin E (21 mg/100 g) and lutein (0.25 mg/100 g). Independently characterized *Moringa oleifera* seed oils have likewise been reported to be oleic acid-rich and to contain tocopherols and phytosterols [36,37,38]. Importantly, plant part, origin, cultivar, and extraction procedure can influence oil composition [37,38]; because those provenance variables were not retained for the present material, the literature profiles are used only for contextual comparison and are not assumed to be identical to the administered oil.

Potential chemical contributors should therefore be discussed cautiously. Tocopherols can interrupt lipid peroxidation chain reactions, and a monounsaturated fatty acid-rich matrix has fewer oxidizable double bonds than a polyunsaturated-rich matrix; these features may be compatible with the lower MDA concentrations observed here. However, individual fatty acids (including oleic acid), tocopherol homologues, phytosterols, phenolics, and isothiocyanates were not quantified in the study material. Moreover, reports of NF-κB suppression and Nrf2 activation by isothiocyanate-enriched Moringa seed extracts [20,21] concern chemically distinct preparations and do not demonstrate that those constituents or pathways mediated the effects of the fixed oil used here. The present data are therefore most appropriately interpreted as evidence of a composite treatment-associated effect of the characterized oil matrix, while constituent-level causality remains to be tested by targeted profiling and mechanistic experiments.

### 4.3. Microbiological, Clinical, Survival, and Histopathological Findings

In the CLP group, S. aureus positivity reached 87.5%, with a recoverable count of 1.69 ± 0.85 log10 CFU/mL, whereas E. coli positivity reached 75%, with a recoverable count of 2.10 ± 0.45 log10 CFU/mL. MOO treatment was associated with lower recoverable bacterial burden, and both organisms were below the detection limit in the 200 mg/kg group. This result does not establish direct antibacterial activity of the oil: no MOO-only in vitro assay, minimum inhibitory or bactericidal concentration, time-kill analysis, or tissue pharmacokinetic assessment was performed. Reduced recovery may reflect a direct effect, improved host containment, preservation of tissue barriers, modulation of inflammation, or a combination of these processes. Accordingly, the finding is reported as reduced recoverable bacterial burden under the experimental conditions rather than proof of bactericidal action.

The observation of lethargy, piloerection, anorexia, hypothermia, reduced locomotor activity, diarrhea, and increased pain score in the CLP group indicates that the model produced a severe clinical picture of sepsis. In the MOO 100 and 200 mg/kg groups, clinical scores decreased to 1.8 ± 0.3 and 1.0 ± 0.2, respectively. Improvements in behavior, pain score, stool consistency, and activity suggest that MOO reduced the overall severity of clinical illness. These clinical improvements were consistent with the reductions in inflammatory and oxidative stress markers, the decrease in recoverable bacterial burden, and the attenuation of histopathological injury. Similarly, previous studies have reported that suppression of inflammation and oxidative stress is accompanied by clinical and histopathological improvement in experimental sepsis models [39].

The 24 h survival rates were 100% in the Control and Sham groups, 62.5% in the CLP group, 87.5% in the MOO 100 mg/kg group, and 100% in the MOO 200 mg/kg group. The higher 24 h survival observed in the high-dose MOO group was accompanied by lower clinical scores, reduced recoverable bacterial burden, increased Apelin and Omentin-1 levels, decreased IL-6, TNF-α, and MDA levels, and better organ histology. These findings indicate a multidimensional improvement in the MOO-treated groups. However, because each group included only eight rats and the follow-up period was limited to 24 h, this finding should not be interpreted as evidence of long-term mortality benefit.

The presence of parenchymal degeneration, inflammatory cell infiltration, and vacuolar injury in the heart, lung, liver, and kidney tissues of the CLP group confirms sepsis-related multi-organ injury. The reduction in these lesions following MOO administration, particularly in the 200 mg/kg group, supports the organ-protective potential of the oil. Sepsis-related organ injury develops through the combined effects of cytokine release, increased endothelial permeability, microcirculatory dysfunction, mitochondrial impairment, and ROS accumulation. Similarly, Koc et al. [39] reported that histopathological lesions in the kidney, lung, and liver were reduced following anti-inflammatory and antioxidant treatment. The parenchymal degeneration, vacuolar injury, and inflammatory infiltration observed in the liver are compatible with sepsis-related hepatic involvement. Ebrahem et al. [40] reported that a Moringa-containing nanoparticle formulation reduced IL-6 levels and histopathological liver damage. Although the experimental model used in that study differed from the present model, their results support the hepatoprotective potential of Moringa. However, because ALT, AST, bilirubin, and albumin levels were not measured in the present study, only histopathological preservation can be discussed, and functional hepatic improvement cannot be confirmed. The parenchymal and vacuolar injury observed in the kidney is compatible with sepsis-associated acute kidney injury. Aksoy et al. [41] reported increased renal MDA and myeloperoxidase activity in a CLP model, while Koc et al. [39] reported tubular degeneration, hyaline casts, and interstitial hyperemia. In the present study, the better preservation of renal histology in the 200 mg/kg MOO group was consistent with reduced cytokine and MDA levels. Sumandjar et al. [42] reported that Moringa reduced MDA levels but did not consistently improve renal necrosis. This discrepancy may be related to differences in extract type, dose, duration of administration, and model severity. Because creatinine, urea, and urine output were not measured in the present study, functional renal recovery could not be evaluated. The degeneration and inflammatory infiltration observed in the lung are compatible with acute lung injury. Koc et al. [39] reported septal thickening and perivascular and peribronchiolar inflammation in an LPS model. Qi et al. [33] and Zhou et al. [34] showed that Omentin-1 reduces pulmonary inflammation, capillary leakage, NF-κB activation, and oxidative stress, thereby preserving endothelial barrier function. These findings support the coexistence of increased Omentin-1 levels with reduced lung injury, IL-6, TNF-α, and MDA levels in the present study. However, direct mediation by Omentin-1 was not demonstrated.

The parenchymal degeneration, inflammatory infiltration, and vacuolar injury observed in the heart are compatible with septic myocardial involvement. Histopathological preservation in the MOO 200 mg/kg group was parallel to increased serum Apelin levels and reduced inflammatory and oxidative stress markers. However, because troponin, CK-MB, electrocardiography, blood pressure, ejection fraction, and Apelin/APJ tissue expression were not measured, functional cardioprotection or direct Apelin-mediated protection cannot be claimed. Overall, the 200 mg/kg MOO dose produced more pronounced biochemical, microbiological, clinical, and histopathological improvement than the 100 mg/kg dose. This finding is consistent with studies suggesting that the biological activity of Moringa compounds may increase with concentration [20,43]. Nevertheless, because only two doses were tested and no formal trend analysis was performed, the optimal dose, minimum effective dose, and toxicity threshold could not be determined. Therefore, the higher dose produced greater effects under the tested conditions; a formal dose–response relationship cannot be established.

Histological preservation should not be equated with functional recovery. Serum ALT, AST, bilirubin, albumin, creatinine, urea, cardiac troponin, and CK-MB; urine output; hemodynamic or echocardiographic variables; and pulmonary gas exchange indices were not measured. Therefore, functional recovery of the liver, kidney, heart, or lung cannot be inferred from morphology alone.

### 4.4. Strengths, Mechanistic Boundaries, and Limitations

A strength of the present study is the integrated assessment of adipokines, inflammatory cytokines, MDA, recoverable bacterial burden, clinical severity, early survival, and multi-organ histology in the technically demanding CLP model. Nevertheless, the results are limited to a 24 h endpoint and eight animals per group. The study lacked a non-septic MOO-only group, an antibiotic comparator arm, longitudinal sampling, direct antibacterial assays, and comprehensive redox measurements. In addition, the retained records did not include authenticated plant part documentation, geographical/commercial origin, voucher specimen, batch number, or extraction/manufacturing procedure, and the analytical panel quantified fatty acid classes rather than individual fatty acids, tocopherol homologues, sterols, phenolics, or isothiocyanates. These limitations preclude constituent-specific attribution and reduce material reproducibility. Future work should use botanically authenticated and batch-defined oil, report the extraction process, perform targeted compositional profiling, include MOO-only and standard-of-care comparator groups, and test candidate pathways together with longer-term survival and organ function outcomes.

Additional limitations affecting interpretation and transparency include the single-sex design, the absence of formal dose trend analysis, missing functional organ injury endpoints, the absence of recoverable pixel-to-micrometer calibration metadata for Figure 2, and the absence of verifiable individual-animal values in the retained revision records. Only female rats were studied, which limits generalizability to males. The missing calibration metadata precluded scientifically valid retrospective scale bars, while the lack of verified individual observations precluded distribution and outlier visualization. Future studies should include both sexes with prespecified sex-stratified analyses, additional doses with appropriate trend modeling, functional organ endpoints, calibrated photomicrographs, and prospective retention and reporting of animal-level data.

## 5. Conclusions

This study showed that *Moringa oleifera* oil (MOO) was associated with beneficial biochemical, microbiological, clinical, and histopathological changes in a CLP-induced experimental sepsis model. The 200 mg/kg MOO dose was associated with a marked reduction in recoverable S. aureus and E. coli counts, with both microorganisms remaining below the detection limit under the conditions of the present microbiological analysis. In the same group, the 24 h survival rate was 100%, compared with 62.5% in the untreated CLP group. MOO administration, particularly at 200 mg/kg, increased serum Apelin and Omentin-1 levels and reduced IL-6, TNF-α, and MDA levels; these changes are consistent with attenuation of sepsis-associated inflammatory activation and lipid peroxidation. Clinical septic signs, including lethargy, reduced activity, hypothermia, pain response, and diarrhea, were also less severe in the MOO-treated groups. Histopathological examination further showed that MOO treatment was associated with reduced parenchymal degeneration, inflammatory cell infiltration, vascular alterations, and vacuolar injury in the liver, kidney, heart, and lung tissues. Overall, these findings suggest that 200 mg/kg MOO may have multi-target protective potential in CLP-induced experimental sepsis by modulating adipokine-related responses, inflammatory activation, oxidative stress, recoverable bacterial burden, and tissue injury. However, because this study was limited to a 24 h follow-up period and did not include a non-septic MOO-only group, additional redox endpoints, direct bactericidal assays, or molecular pathway analyses, the present findings do not establish an intrinsic antioxidant or anti-inflammatory mechanism. Further studies with larger sample sizes, longer observation periods, standardized batch-defined oil preparations, and targeted molecular validation are required before definitive therapeutic conclusions can be drawn.

## Figures and Tables

**Table 1:** Effects of CLP and different doses of MOO on serum apelin, omentin-1, IL-6, TNF-α, and MDA levels in experimental sepsis model.

Treatment Group	Apelin (ng/mL)	Omentin-1 (ng/mL)	IL-6 (pg/mL)	TNF-α (pg/mL)	MDA (nmol/mL)
Control (*n* = 8)	0.45 ± 0.04 ^a^	32.1 ± 2.5 ^d^	12.2 ± 1.1 ^d^	14.8 ± 1.5 ^d^	1.7 ± 0.2 ^d^
Sham (*n* = 8)	0.43 ± 0.05 ^a^	31.8 ± 2.2 ^d^	12.8 ± 1.3 ^d^	15.5 ± 1.7 ^d^	1.9 ± 0.3 ^d^
CLP (*n* = 8)	0.42 ± 0.05 ^a^	12.4 ± 1.5 ^a^	45.2 ± 3.1 ^a^	58.6 ± 4.2 ^a^	6.8 ± 0.5 ^a^
CLP + MOO 100 mg/kg (*n* = 8)	0.68 ± 0.08 ^b^	18.6 ± 2.2 ^b^	32.1 ± 2.4 ^b^	42.3 ± 3.5 ^b^	4.2 ± 0.4 ^b^
CLP + MOO 200 mg/kg (*n* = 8)	1.15 ± 0.12 ^c^	28.4 ± 3.1 ^c^	18.4 ± 1.8 ^c^	24.1 ± 2.8 ^c^	2.5 ± 0.3 ^c^
*p*-value	<0.001	<0.001	<0.001	<0.001	<0.001

Data are presented as mean ± standard deviation. Within each column, means with different superscript letters differ significantly (*p* < 0.05). Group comparisons were performed using one-way analysis of variance followed by Duncan’s multiple range test. CLP, cecal ligation and puncture; MOO, *Moringa oleifera* oil; IL-6, interleukin-6; TNF-α, tumor necrosis factor-α; MDA, malondialdehyde.

**Table 2:** Recoverable *Staphylococcus aureus* and *Escherichia coli* counts in blood and peritoneal samples from the experimental groups.

Treatment Group	Bacterial Species	Positivity, *n*/N	Positivity (%)	Recoverable Bacterial Count (log10 CFU/mL)	*p*-Value
Control	*S. aureus*	0/8	0	<1.00	—
Control	*E. coli*	0/8	0	<1.00	—
Sham	*S. aureus*	1/8	12.5	1.15 ± 0.30	>0.05
Sham	*E. coli*	0/8	0	<1.00	—
CLP	*S. aureus*	7/8	87.5	1.69 ± 0.85	—
CLP	*E. coli*	6/8	75	2.10 ± 0.45	—
CLP + MOO 100 mg/kg	*S. aureus*	1/8	12.5	<1.00	<0.001
CLP + MOO 100 mg/kg	*E. coli*	0/8	0	<1.00	<0.001
CLP + MOO 200 mg/kg	*S. aureus*	0/8	0	<1.00	<0.001
CLP + MOO 200 mg/kg	*E. coli*	0/8	0	<1.00	<0.001

Data are presented as the mean ± standard deviation where applicable. Values below 1.00 log10 CFU/mL were considered below the detection limit. *p*-values represent comparisons with the untreated CLP group. No direct bactericidal, minimum inhibitory concentration, minimum bactericidal concentration, or time-kill assay was performed. CLP, cecal ligation and puncture; MOO, *Moringa oleifera* oil; CFU, colony-forming units.

**Table 3:** Clinical, behavioral, and 24 h survival findings in the experimental groups.

Treatment Group	Behavioral Score (0–5)	Clinical Score (0–4)	Surviving Animals, *n*/N	24 h Survival (%)	Grimace Pain Score	Stool Consistency
Control (*n* = 8)	0 (Normal)	0.0 ± 0.0 ^d^	8/8	100	0	Normal
Sham (*n* = 8)	0 (Normal)	0.2 ± 0.1 ^d^	8/8	100	0	Normal
CLP (*n* = 8)	4 (Severe sepsis)	3.8 ± 0.5 ^a^	5/8	62.5	3	Diarrheic
CLP + MOO 100 mg/kg (*n* = 8)	2 (Mild lethargy)	1.8 ± 0.3 ^b^	7/8	87.5	2	Soft
CLP + MOO 200 mg/kg (*n* = 8)	1 (Active)	1.0 ± 0.2 ^c^	8/8	100	1	Normal
Overall *p*-value for clinical score	—	<0.001	—	—	—	—

Data are presented as the mean ± standard deviation where applicable. Within the clinical score column, means with different superscript letters differ significantly (*p* < 0.05). Clinical scores were compared using one-way analysis of variance followed by Duncan’s multiple range test. Survival is reported descriptively as *n*/N and percentage. Behavioral score, Grimace pain score, and stool consistency are descriptive. CLP, cecal ligation and puncture; MOO, *Moringa oleifera* oil.

## Data Availability

The aggregate data supporting the findings are contained within the article. Verifiable individual-animal values were not available in the retained revision records; accordingly, individual-data plots were not generated from reconstructed observations.

## Abbreviations

CLP, cecal ligation and puncture; MOO, *Moringa oleifera* oil; IL-6, interleukin-6; TNF-α, tumor necrosis factor-α; MDA, malondialdehyde; CFU, colony-forming units; ELISA, enzyme-linked immunosorbent assay; H&E, hematoxylin and eosin; HPLC-UV/FLD, high-performance liquid chromatography with ultraviolet/fluorescence detection.
